# FMO family may serve as novel marker and potential therapeutic target for the peritoneal metastasis in gastric cancer

**DOI:** 10.3389/fonc.2023.1144775

**Published:** 2023-05-18

**Authors:** Xumeng Gong, Dong Hou, Shengning Zhou, Jianan Tan, Guangyu Zhong, Bing Yang, Lang Xie, Fanghai Han, Lin Zhong

**Affiliations:** ^1^ Department of Surgical Oncology, Yuebei People’s Hospital, Shaoguan, Guangdong, China; ^2^ Department of Head-Neck and Breast Surgery, Yuebei People’s Hospital of Shantou University, Shaoguan, Guangdong, China; ^3^ Department of Gastrointestinal Surgery, Sun Yat-sen Memorial Hospital, Sun Yat-sen University, Guangzhou, Guangdong, China; ^4^ Department of General Surgery, Zhujiang Hospital, Southern Medical University, Guangzhou, Guangdong, China

**Keywords:** FMO family, peritoneal metastasis, gastric cancer, novel marker, therapeutic target

## Abstract

**Objective:**

To explore the relationship between flavin-containing monooxygenases (FMOs) and peritoneal metastasis (PM) in gastric cancer (GC).

**Materials and methods:**

TIMER 2.0 was used to perform pan-cancer analysis and assess the correlation between the expression of FMOs and cancers. A dataset from The Cancer Genome Atlas (TCGA) was used to analyze the correlation between FMOs and clinicopathological features of GC. PM is well established as the most common mode of metastasis in GC. To further analyze the correlation between FMOs and PM of GC, a dataset was obtained from the National Center for Biotechnology Information Gene Expression Omnibus (GEO) database. The results were validated by immunohistochemistry. The relationship between FMOs and PM of GC was explored, and a novel PM risk signature was constructed by least absolute shrinkage and selection operator (LASSO) regression analysis. The regression model’s validity was tested by multisampling. A nomogram was established based on the model for predicting PM in GC patients. The mechanism of FMOs in GC patients presenting with PM was assessed by conducting Gene Ontology (GO) and Kyoto Encyclopedia of Genes and Genomes (KEGG) analyses in TCGA and GEO datasets. Finally, the potential relationship between FMOs and immunotherapy was analyzed.

**Results:**

The pan-cancer analysis in TCGA and GEO datasets showed that FMO1 was upregulated, while FMO2 and FMO4 were downregulated in GC. Moreover, FMO1 and FMO2 correlated positively with the T and N stage of GC in the TCGA dataset. FMO1 and FMO2 expression was a risk factor for GC (hazard ratio: 1.112 and 1.185). The overexpression of FMO1 was significantly correlated with worse disease-free-survival (DFS) and overall survival (OS). However, no relationship was found between FMO2 expression in GC and DFS and OS. PM was highly prevalent among GC patients and typically associated with a worse prognosis. FMO1 was highly expressed in GC with PM. FMO1 and FMO2 were positively correlated with PM in GC. We identified a 12-gene panel for predicting the PM risk signature by LASSO (Area Under Curve (AUC) = 0.948, 95%CI: 0.896–1.000). A 10-gene panel for PM prediction was identified (AUC = 0.932, 95%CI: 0.874–0.990), comprising FMO1 and FMO2. To establish a model for clinical application, a 7-gene panel was established (AUC = 0.927, 95% CI: 0.877–0.977) and successfully validated by multisampling. (AUC = 0.892, 95% CI: 0.878–0.906). GO and KEGG analyses suggest that FMO1 and FMO2 regulate the extracellular matrix and cell adhesion. FMO1 and FMO2 were positively correlated with the immune score of GC, and their expression was associated with the infiltration of immune cells.

**Conclusion:**

PM in GC is strongly correlated with FMOs. Overall, FMO1 and FMO2 have huge prospects for application as novel diagnostic and therapeutic targets.

## Introduction

Peritoneal metastasis (PM) is the most common mode of metastasis in gastric cancer (GC) (1), with 20%–30% of GC patients reported to have PM at initial presentation (2) and 40%–50% sustaining relapse due to PM (3). PM in GC has been associated with serious complications, such as malignant ascites (4) and ileus (5). PM of GC is usually rapidly fatal, with a median survival time of less than 1 year, mainly due to the lack of effective treatment (6). The effectiveness of traditional chemotherapy is poor because of the blood–peritoneal barrier (7). The prognosis of PM in GC can be improved to a certain extent by cytoreductive surgery (CRS) combined with hyperthermic intraperitoneal chemotherapy (HIPEC). CRS involves resecting cancer that has spread to the peritoneal cavity, while HIPEC needs specialized equipment (1). It is widely thought that targeted therapy can effectively improve the prognosis of advanced GC, representing a potential therapeutic modality for PM in GC (8).

With a better understanding of the molecular mechanisms of GC, targeted therapy has gained substantial attention to improve the prognosis and reduce toxicity (9). Clinical trials have shown that targeted therapy can improve overall survival (OS) and DFS in partial GC patients (1). Targeted therapy has been considered an important treatment for advanced GC. Nonetheless, there are strict requirements for targeted therapy in GC, such as human epidermal growth factor receptor-2 (HER-2) positivity, vascular endothelial growth factor receptor (VEGFR) positivity, and programmed cell death-ligand 1 (PD-L1) overexpression (10). Identifying new targets in GC is of great significance in improving the prognosis of this patient population, emphasizing the need for further research.

Flavin-containing monooxygenases (FMOs) and cytochromes P450(CYP450) are the most important oxygenases in humans (11, 12). This enzyme system is mainly involved in the biotransformation of drugs and exogenous substances (13). The enzymatic activity is modulated by extrinsic factors and regulated by many cytokines, such as interleukin-6 (IL-6) and tumor necrosis factor α (TNF-α) (14). These cytokines are important components of the tumor microenvironment (TME). As phase I metabolic enzymes, FMOs and CYP450 produce ROS in the process of biotransformation (15, 16). The TME and ROS are related to the development of cancer. Recent research has demonstrated that FMOs and CYP450 are associated with the growth and treatment of many cancers.

The FMO gene family comprises 11 genes; FMO1, FMO2, FMO3, FMO4. and FMO5 are functional genes, and the remaining family members are pseudogenes (17). It has been established that FMOs are expressed in many cancers and are associated with the development and prognosis. FMO1 serves as a predictor of RFS in papillary thyroid cancer (18). FMO2 is significantly upregulated in early-stage oral squamous cell carcinoma (19). Moreover, it has been reported that miR205-5p may play a significant role in the prognosis of neck squamous cell carcinoma, and FMO2 is the target gene (20). Furthermore, FMO4 shapes immuno-metabolic reconfiguration in hepatocellular carcinoma (21). Last but not least, FMO5 is associated with a poor prognosis in colorectal cancer (22).

Inspired by these findings on the relationship between FMOs and cancers, we explored the correlation between FMOs and GC. We revealed a hitherto undocumented correlation between FMOs and PM in GC. Overall, FMOs represent a potential target for diagnosing and treating PM in GC.

## Materials and methods

### Data acquisition and expression analysis

Transcriptome data on pan-cancer and GC tissues were collected from the Cancer Genome Atlas (TCGA) (https://portal.gdc.cancer.gov) and normalized using the fragments per kilobase of exon per million fragments mapped (FPKM) method using R. (version 4.2.1). The ‘limma’ package was used to evaluate changes in the expression levels of other members of the FMO family except pseudogenes between tumor samples and normal samples in pan-cancer, and the ‘ggplot’ tool was used to visualize the results. A *P*-value < 0.05 was statistically significant. To further investigate PM in GC, the Gene Expression Omnibus (GEO) dataset GSE15081 was obtained from the National Center for Biotechnology Information database (https://www.ncbi.nlm.nih.gov), and the sequencing data of 77 patients without PM and 31 patients with PM were included for subsequent analysis.

### Correlation analysis of clinicopathological features

Similarly, clinical information from TCGA was retrieved for patients in the GC cohort, including age, gender, TNM stage, grade, survival status, and survival time. FMO1, FMO2, FMO3, FMO4, and FMO5 gene expression differences in distinct clinicopathological characteristics were investigated using the ‘ggpubr’ and ‘limma’ packages. A box plot was generated to visualize the statistical significance of the differences (significance identification: *ns*, *p* 0.05; *, *p* 0.05; * *, *p* 0.01; * * *, *p* 0.001). The ‘ComplexHeatmap’ software was used to summarize and illustrate all clinicopathological characteristics and gene expression levels. The ‘ComplexHeatmap’ software summarized and illustrated all clinicopathological characteristics and gene expression levels. Finally, univariate and multivariate cox regression analyses based on gene expression levels and clinical features were conducted to determine whether the FMO genes were independent prognostic factors.

### Analysis of survival

The ‘ survminer ‘ programming package in R software was loaded, and the median of each gene expression was selected as the best cutoff value. The patient cohort was divided into high-expression and low-expression groups. The Kaplan–Meier survival curve assessed the differences in overall survival (OS) and progression-free survival (PFS) between the two groups to better understand the relationship between FMO genes and the GC prognosis.

### Peritoneal metastasis correlation analysis

The ‘limma’ package was used to compare the peritoneal metastatic group to the non-PM group in the GSE15081 dataset. The screening criteria were logFC > 0, *P* 0.05. FMO1, FMO2, FMO3, FMO4, and FMO5 gene expression for each sample in the dataset were sorted out. Spearman correlation analysis was employed to investigate the relationship between each gene and the incidence of PM.

### Immunohistochemistry

To validate the expression of FMO1 and FMO2 in non-PM-GC and PM-GC patients, immunohistochemistry (IHC) was performed. The study was performed in 10 non-PM-GC and 10 PM-GC tissues. At last, for the FMO2 group, valid results were obtained in nine non-PM-GC and nine PM-GC. The experimental procedure and statistical methods were operated as our previous report (23). The antibodies against the FMO1and FMO2 were used with source and dilution ratios indicated: FMO1(CusaBio, #CSB-PA08746, 1:100); FMO2 (Affinity Biosciences, #DF16081, 1:100). The score of expression is divided into four grades, high positive (score = 4), positive (score =3), low positive (score = 2), and negative (score = 1).

### Identifying peritoneal metastasis–related genes and developing a prediction model

Finally, genes reported in the literature were integrated with the above FMO family gene correlation analysis results and GSE15081 dataset mRNA expression. The study comprised 26 genes (C10orf95, CHCHD3, CKB, CTF1, CYP2W1, FIBP, FMO1, FMO2, GDNF, GRPEL1, KCNJ6, LMBR1, LTBP3, MC5R, PHYHD1, POPDC2, PROK1, RNF186, RPSA, SLITRK6, STRBP, STT3B, TXN, WDR48, ZBTB1, and ZIC3). The least absolute shrinkage and selection operator (LASSO) regression analysis was used to further select genes associated with PM for inclusion in the model, and the logistics regression model was built using the ‘glm’ function. To build a receiver operating characteristic (ROC) curve, the ‘pROC’ package was used. Finally, a nomogram was generated to illustrate the model’s predictive performance objectively, and calibration curve analysis was conducted to assess the consistency index and its accuracy.

### Analysis of functional enrichment

Patients in the cancer genome atlas- stomach adenocarcinoma (TCGA-STAD) cohort were split into high- and low-expression groups based on the median expression level of the FMO1 and FMO2 genes. The R package limma was used to identify differentially expressed genes (DEGs) with logFC > 1 and FDR 0.05 as screening criteria.

### Flavin-containing monooxygenase gene–related immune microenvironment analysis

The Estimation of STromal and Immune cells in MAlignant Tumor tissues using Expression data (ESTIMATE) algorithm was used to determine the scores of stromal cells and immune cells in the tissues of TCGA GC patients, and differential expression analysis was conducted based on gene expression levels. The tumor purity and immunological score were evaluated between the high- and low-expression groups of each gene in the FMO gene family. The fraction of cell types in the mixed cell population was determined using the CIBERSORT software to quantify the infiltration abundance of 22 immune cells in GC tissues. The R software packages ‘vioplot’ and ‘ggpubr’ were used to visualize the abundance expression of immune infiltrating cells in high- and low-expression groups. Using the ‘GSVA’ software tool, a box plot was generated to visualize the variations in immune function between the high- and low-expression groups.

## Results

### The expression of flavin-containing monooxygenases is correlated with the prognosis of gastric cancer

**Figure 1:**
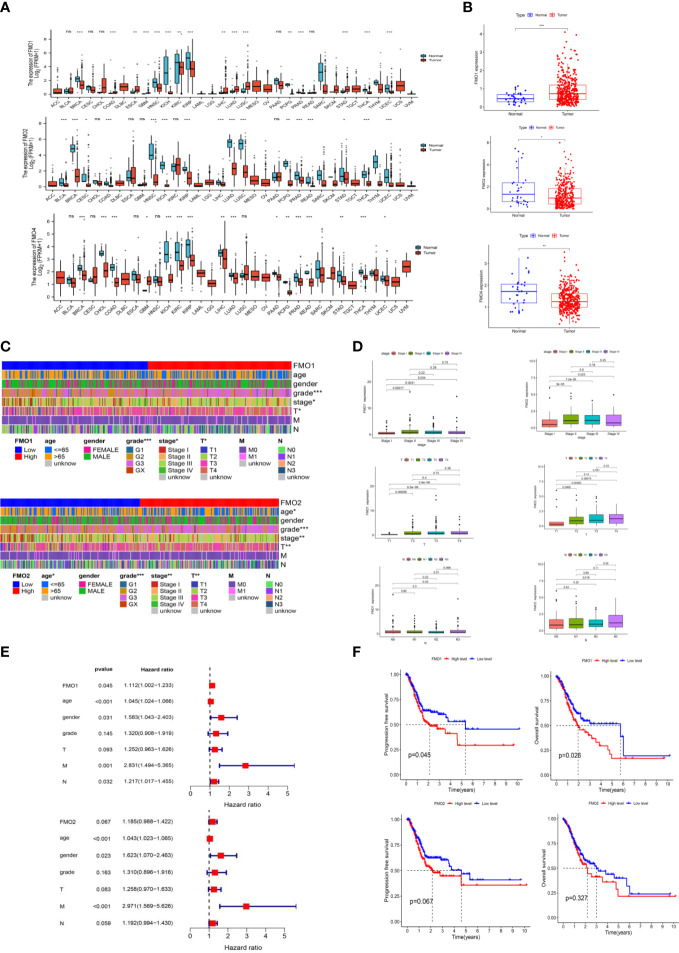
The expression of flavin-containing monooxygenases (FMOs) is correlated with the prognosis of gastric cancer (GC). **(A)** The expression of FMOs in different cancers from The Cancer Genome Atlas (TCGA) data analyzed. **(B)** FMO expression in normal and tumor tissues in GC from TCGA data. **(C)** Heatmap of the relationship between FMO1 and FMO2 expression and clinicopathological analysis. **(D)** Association between FMO1 and FMO2 expression and the pathological, T, and N stages of GC. **(E)** multivariate Cox hazard regression analysis for overall survival in GC. **(F)** Increased FMO1 expression predicted a worse prognosis in GC; there was no significant correlation between FMO2 and the prognosis in GC. *p < 0.05, **p < 0.01, ***p < 0.001.

FMO overexpression has been associated with a poor prognosis and is a potential therapeutic target in many cancers, such as colorectal cancer (22), papillary thyroid cancer (18), and hepatocellular carcinoma (21). To explore the prognostic value of FMOs, systematic pan-cancer analysis was performed by TIMER2.0 (http://timer.cistrome.org/). The FMOs were differentially expressed between most cancers and normal tissues. FMO1 was overexpressed in GC, while FMO2 and FMO4 were low expressed (**Figure 1A**). Subsequently, we evaluated the expression level of FMOs in TCGA, and the results were consistent with pan-cancer analysis (**Figure 1B**).

To examine the correlation between the expression of FMOs and the clinicopathological characteristics, including age, gender, grade, stage, and TNM stage in GC, we further analyzed the dataset from TCGA. Based on the median values of the FMO1 or FMO2 mRNA level, the GC patients were classified into high- and low-expression groups. The results demonstrated that FMO1 and FMO2 are associated with the clinicopathological characteristics of GC, while there was no significant relationship between the expression of FMO4 and clinicopathological characteristics (**Figures 1C**; **S1C**). FMO1 is significantly associated with tumor grade, stage, and T stage, while FMO2 is correlated with age, grade, stage, and T stage (**Figure 1C**).

The association between FMO1 and FMO2 expression in GC with clinicopathological characteristics was further investigated. As shown in **Figure 1D**, FMO1 and FMO2 were upregulated in patients with advanced-stage disease compared to stage I. Increased expression of FMO1 and FMO2 correlated significantly with the T stage (*p* < 0.01). Furthermore, increased FMO2 expression in GC was significantly associated with a high N stage (N3 vs. N0, *p =* 0.019).

The prognostic value of FMO1 and FMO2 in GC was analyzed. As indicated in **Figure 1E**, the overexpression of FMO1 was significantly related to a worse prognosis in GC [hazard ratio (HR) =1.112, *p =* 0.045], while FMO2 had no significant effect on prognosis (HR = 1.185, *p =* 0.067). To further explore the effects of FMO1 and FMO2 on the survival in GC, the Kaplan–Meier curves of DFS and OS were generated (**Figure 1F**). FMO1 expression was significantly correlated with DFS (*p =* 0.045) and OS (*p =* 0.026). However, FMO2 exhibited no significant prognostic value for DFS (*p =* 0.067) or OS (*p =* 0.327). Therefore, FMO1 and FMO2 expression is associated with the clinicopathological characteristics and prognosis in GC.

### The expression of flavin-containing monooxygenases is correlated with peritoneal metastasis in gastric cancer

The peritoneum is the main site of metastasis in advanced GC. Although the exact mechanism of PM in GC is unclear, the “seed and soil” hypothesis has been accepted as the most common hypothesis of PM (24). According to this theory, cancer cells shed from the primary tumor are crucial for PM development. The T stage is used to assess the levels of invasion in GC. Serosal invasion (T4) has been closely associated with PM in GC (25).

**Figure 2:**
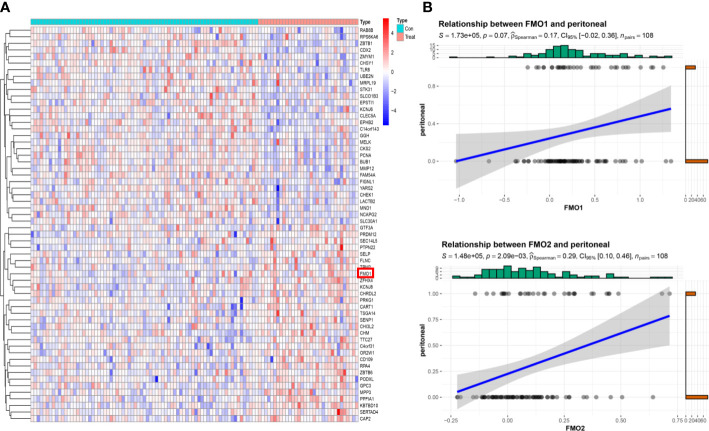
The expression of FMOs is correlated with peritoneal metastasis (PM) in GC. **(A)** The top 30 differentially expressed genes (DEGs) between peritoneal relapse (PR) and without peritoneal relapse (NPR); clustering analysis results are shown in the heatmap. **(B)** The association between FMO1, FMO2, and PR.

The correlation between FMOs and clinicopathological characteristics in GC was subsequently explored. We found that FMO1 and FMO2 expression positively correlated with the T stage in GC. The GSE15081 dataset was used to explore the correlation between FMO1, FMO2, and PM in GC. The GSE15081 dataset contains 33 patients with peritoneal relapse (PR) and 75 patients without peritoneal relapse (NPR). The DEGs were identified between PR and NPR. FMO1 and FMO2 were overexpressed in PR patients. FMO1 was significantly upregulated in the PR group (**Figure 2A**). The correlation between FMO1, FMO2, and PM was evaluated by correlation analysis. The results indicated that FMO1 and FMO2 expression was closely related to PM (**Figures 2B**; **S2**). To validate the results, we performed IHC in non-PM-GC and PM-GC patients` tissue. The results showed that the expression of FMO1 and FMO2 was higher in PM-GC than non-PM-GC (**S3**). Thus, FMO1 and FMO2 may act as potential therapeutic targets.

### Clinical prediction model of peritoneal metastasis in gastric cancer based on flavin-containing monooxygenases

PM is the most common distant metastasis in GC, resulting in serious complications and a poor prognosis. Due to the blood–peritoneal barrier, systemic chemotherapy has limited efficacy in treating PM in GC. Early diagnosis and treatment of PM are the best way to improve the prognosis in GC. Computerized tomography (CT) scans and diagnostic laparoscopy are the main methods to identify PM in GC. However, CT scan exhibits a low sensitivity and specificity for PM diagnosis (26). Diagnostic laparoscopy is the recommended diagnosis method but is invasive, bringing physical and psychological trauma to patients (27). A clinical model based on biomarkers improves sensitivity and specificity and avoids unnecessary invasive procedures.

The GSE15081 dataset was widely used to identify the biomarker for predicting PM in GC. According to the GSE15081 dataset, the genes associated with PR were identified by Takeno et al. in 2010 for the first time (28). The gene list is as follows: RNF186, ZBTB1, RPSA, LMBR1, STRBP, MC5R, C10orf95, FIBP, ZIC3, WDR48, CKB, TXN, CTF1, GRPEL1, LTBP3, CHCHD3, CYP2W1, GDNF, and PROK1. Based on GSE15081, Lee et al. identified a panel of 12 genes containing ZBTB1, CHCHD3, KLHL41, POPDC2, LTBP3, CAVIN2 (SDPR), STT3B, TXNDC16, PHYHD1, KCNJ6, SLITRK6, and LMBR1. A logistic regression model was constructed with the 12 genes, yielding an AUC of 0.95 (CI: 0.89– 0.98) (29). To establish a clinical prediction model based on FMOs, FMO1, FMO2, and the above-mentioned genes were regarded as the target genes.

**Figure 3:**
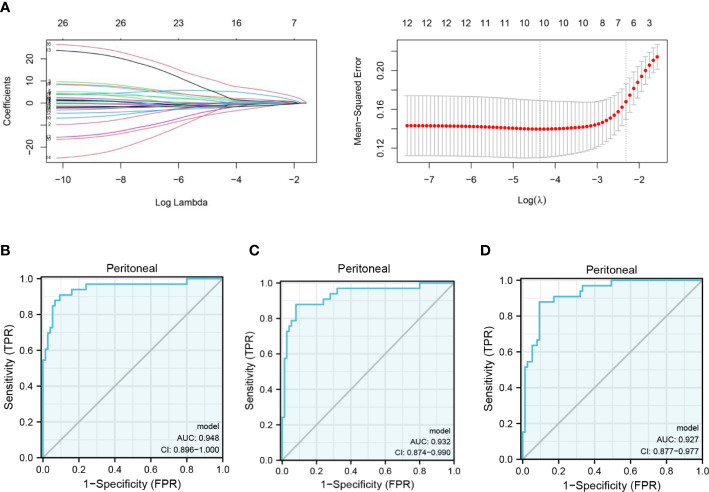
Clinical prediction model of PM in GC based on FMOs. **(A)** The gene signature with PM was constructed by least absolute shrinkage and selection operator regression. **(B)** Receiver operating characteristic (ROC) curve illustrating the performance of the 12-gene panel. **(C)** ROC curve illustrating the performance of the 10-gene panel. **(D)** ROC curve illustrating the performance of the 7-gene panel.

In the GSE15081 dataset, information on KLHL41, CAVIN2 (SDPR), and TXNDC16 was not found. Eventually, C10orf95, CHCHD3, CKB, CTF1, CYP2W1, FIBP, FMO1, FMO2, GDNF, GRPEL1, KCNJ6, LMBR1, LTBP3, MC5R, PHYHD1, POPDC2, PROK1, RNF186, RPSA, SLITRK6, STRBP, STT3B, TXN, WDR48, ZBTB1, and ZIC3 were enrolled in the analysis. The gene signature was established by LASSO regression analysis (**Figure 3A**). A logistic regression model was generated to predict PM in GC incorporating 12 genes (AUC: 0.948, CI: 0.896–1.000) (**Figure 3B**). A 10-gene panel for the prediction of PM was identified by LASSO, including FMO1 and FMO2. According to the 10-gene panel, a model was established (AUC: 0.932, CI: 0.874–0.990) (**Figure 3C**). To facilitate the clinical application of the model, a 7-gene panel was derived by LASSO. We developed a risk prediction model based on the 7-gene panel (AUC: 0.927, CI: 0.877–0.977) (**Figure 3D**) as follows: -2.0336 + 8.7561*ZIC3 + -3.0466*ZBTB1 + 10.0109*CTF1 + 2.2274*POPDC2 + -1.9081*KCNJ6 + 3.2875*FMO2 + -1.931* TXN. We validated the 7-gene panel model by multisampling (AUC = 0.892, 95% CI: 0.878–0.906). Importantly, the model based on FMOs exhibited a satisfactory performance in predicting PM in GC.

### Nomogram was constructed to predict the peritoneal metastasis in gastric cancer

**Figure 4:**
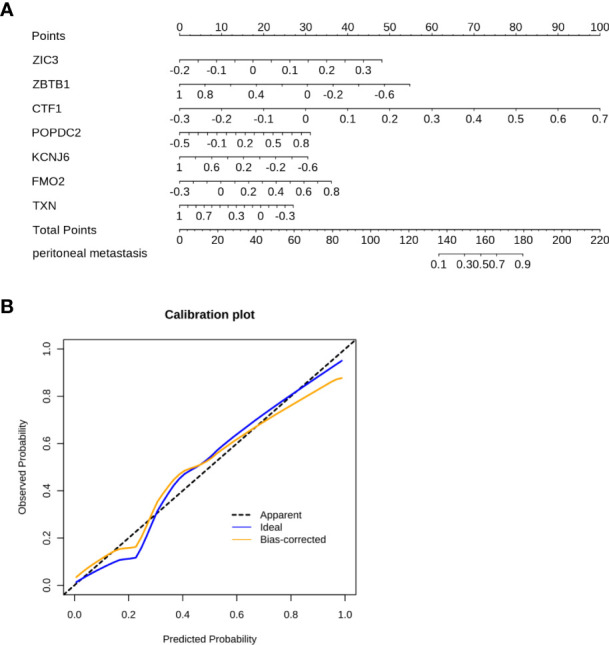
The nomogram was constructed to predict the PM in GC. **(A)** The 7-gene panel-based nomogram for the prediction of PM in GC. (The expression of each gene derives the number of points, the sum of these numbers is the total points, the probability of peritoneal metastasis was determined by the total points.) **(B)** Calibration plots for PM prediction in GC for training and internal validation.

A nomogram represents a convenient tool to evaluate the risk of disease. As shown above, ZIC3, ZBTB1, CTF1, POPDC2, KCNJ6, FMO2, and TXN were included in the prediction model. Based on the risk factors, a comprehensive risk nomogram was developed to predict the PM in GC (**Figure 4A**). Each variable (gene) in the nomogram has a corresponding score on the point scale. After summation of the scores for each gene, the probability of PM can be obtained for individual patients.

The C-index of the nomogram was 0.927 (**Figure 4B**). As shown in **Figure 4B**, the calibration curve showed a good agreement between the predicted and actual outcomes. Accordingly, a nomogram based on FMO2 can help clinicians to predict PM in GC.

### The potential mechanism of flavin-containing monooxygenases regulate peritoneal metastasis in gastric cancer

**Figure 5:**
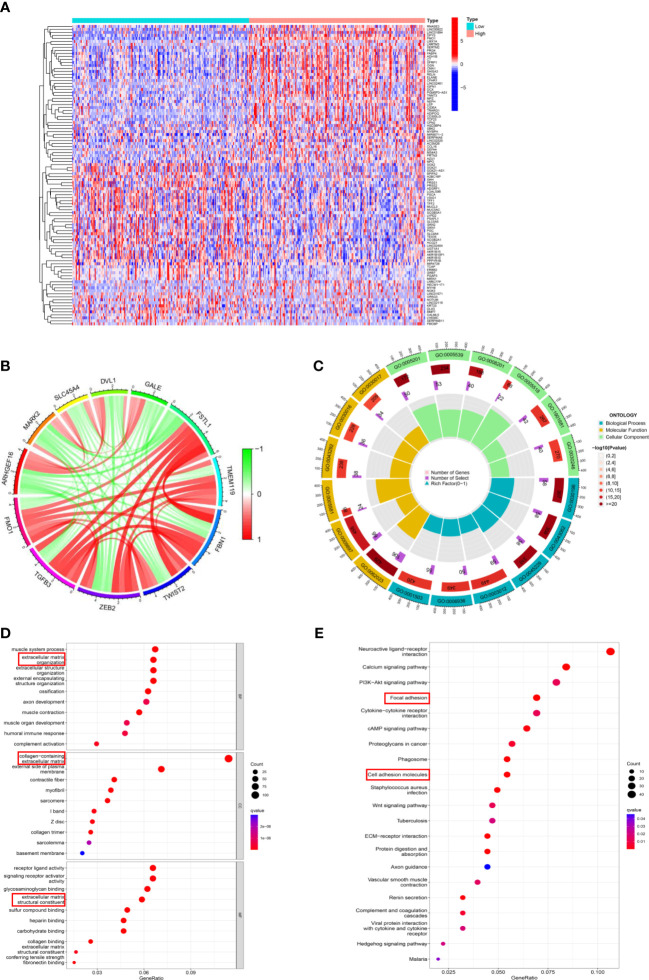
The potential mechanism of FMOs in regulating PM in GC. **(A)** The DEGs between the high FMO1 group and the low FMO1 group visualized in a heatmap. **(B)** Chord diagram constructed based on FMO1 and coexpression genes. The red line indicated positive regulation, while the blue indicated negative regulation. **(C)** Gene Ontology (GO) enrichment analysis was shown by a circle plot. The outermost circle indicates the GO ID, the middle circle indicates the number of genes in the GO term, the innermost circle represents the DEGs in the GO term, and the bar graph of the circle diagram represents the ratio of genes in the GO term. **(D)** GO analysis of the DEGs related to FMO1. The results are shown in a bubble plot. The size of the bubble represents the number of genes, and the color of the bubble represents the adjusted *p*-value. **(E)** Kyoto Encyclopedia of Genes and Genomes analysis of the DEGs related to FMO1. The size of the bubble represents the number of genes, and the color of the bubble represents the adjusted *p*-value.

To elucidate the potential mechanism of FMOs in regulating PM in GC, the DEGs related to FMO1 and FMO2 were identified (**Figures 5A**; **Supplemental S4A**). The relationships between FMO1, FMO2, and related DEGs were analyzed. As shown in **Figure 5B**, FMO1 positively regulated the expression of TGFB3, ZEB2, TWIST2, FBN1, TMEM119, and FSTL1, while the expression of GALE, DVL1, SLC45A4, MARK2, and ARHGEF16 were negatively regulated by FMO1. Moreover, the expression of MOXD1, ANKRD35, HOPX, SLC9A9, BOC, and PALMD were positively regulated by FMO2, while FMO2 negatively regulated the expression of UBE2T, PYCR1, SPC25, EBP, and BIRC5 (**S4B**).

Subsequently, the DEGs underwent Gene Ontology (GO) function and Kyoto Encyclopedia of Genes and Genomes (KEGG) pathways enrichment analyses. The results of FMO1- and FMO2-related GO analyses are shown in **Figures 5C**; **S4C**. FMO1 was significantly enriched in biological process (BP) GO terms, including extracellular matrix organization and extracellular structure organization (**Figure 5D**). Moreover, the DEGs were mainly associated with cell component (CC) GO terms, including a collagen-containing extracellular matrix (**Figures 5D**; **S4D**). For the molecular function (MF), FMO1-related DEGs were mainly enriched in extracellular matrix structural constituents (**Figures 5D**; **S4D**). KEGG pathway analysis showed that FMO1-related genes were associated with focal adhesion and cell adhesion molecules (**Figure 5E**), and the FMO2-related genes were associated with cell adhesion molecules (**Figure S4E**).

### The expression of flavin-containing monooxygenases and immunotherapy

**Figure 6:**
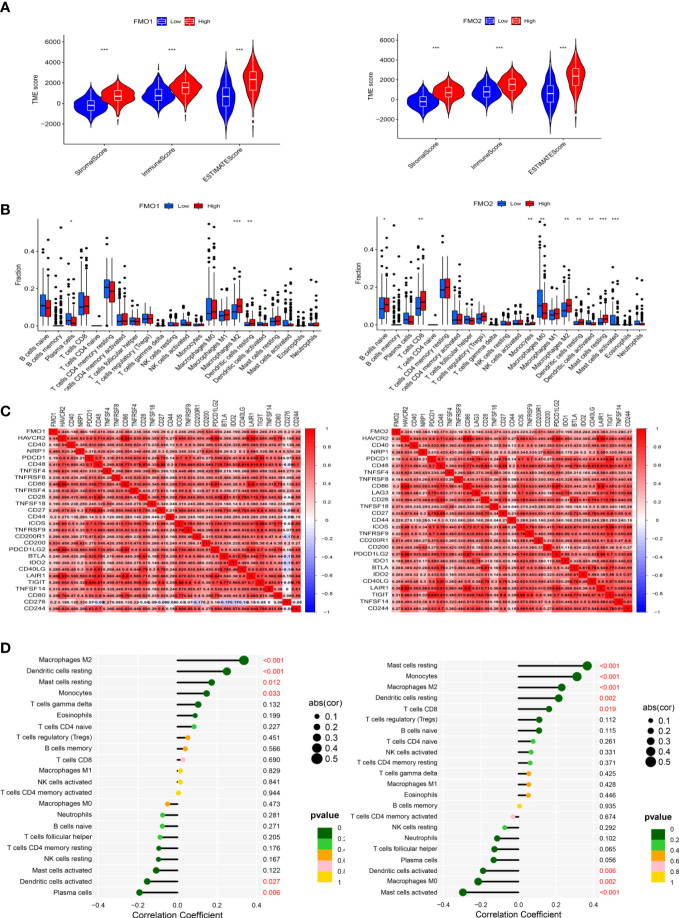
The expression of FMOs and immunotherapy. **(A)** Violin plot of the relationship between the expression of FMOs and immune microenvironment scores in GC. **(B)** Bar plot indicating the difference in 22 types of infiltrated immune cells between the high- and low- expression FMO groups in TCGA. Wilcoxon rank sum was used for the statistical analysis. **(C)** Heatmap showing the correlation between the expression of FMO1 and FMO2 and immune checkpoint genes. **(D)** Lollipop chart showing the correlation between the expression of FMO1 and FMO2 and the 22 infiltrated immune cells. *p < 0.05, **p < 0.01, ***p < 0.001.

PM is the most important metastasis in GC, with no effective treatment currently available. Immunotherapy has been recommended as the third-line treatment of advanced GC by the National Comprehensive Cancer Network (NCCN) and may act as a potentially effective adjuvant therapy for PM in GC (10). To explore the relationship between the expression of FMOs and the TME, the stromal score, immune score, and ESTIMATE score were analyzed in high- and low-FMO expression groups. As shown in **Figure 6A**, the stromal, immune, and ESTIMATE scores were significantly higher in the patients with a high expression of FMO1 and FMO2. We hypothesized that the expression of FMO1 and FMO2 is correlated with the infiltration of immune cells. The association between FMO1, FMO2, and 22 types of immune cells was subsequently investigated. We found that FMO1 expression was positively correlated with M2 macrophages, resting dendritic cells, and monocytes and negatively correlated with activated dendritic cells and plasma cells. In contrast, FMO2 expression was positively correlated with resting mast cells, monocytes, M2 macrophages, resting dendritic cells, and CD8T cells and negatively correlated with activated dendritic cells, M0 macrophages, and activated mast cells (**Figure 6D**).

To further explore the relationship between the expression of FMO1 and FMO2 and the immune cells, the difference and correlation analyses were conducted. We found that FMO1 and FMO2 expression was associated with three and nine types of tumor-infiltrating immune cells, respectively (**Figure 6B**). Plasma cells were negatively correlated with FMO1 expression, while M1 macrophages and resting dendritic cells were positively correlated with the expression of FMO1. In contrast, naive B cells, CD8 T cells, monocytes, M2 macrophages, resting dendritic cells, and resting mast cells were positively correlated with the expression of FMO2, while M0 macrophages, activated dendritic cells, and activated mast cells were negatively correlated with FMO2 expression.

Immune checkpoint blockade therapy has huge prospects for anticancer immunotherapy. The relationship between the expression of FMO1, FMO2, and immune checkpoint molecules was analyzed. The results showed that FMO1 expression was positively correlated with CD200, NRP1, and CD86, while FMO2 was positively correlated with CD200R1, NRP1, and CD49 (**Figure 6C**).

## Discussion

Distant metastases remain the leading cause of GC-related death worldwide. The peritoneal cavity has been established as the most common site of distant metastasis in GC (6). Accordingly, PM has become the major cause of GC-related death. Once PM is diagnosed, the median survival time of GC is only 5 months (7). Worse, PM is often associated with serious complications, and the patient quality of life is extremely poor. Unfortunately, there is no effective therapeutic strategy for treating PM in GC. Due to the blood–peritoneal barrier, systemic chemotherapy is ineffective in treating PM (30). Importantly, cytoreductive surgery (CRS) + hyperthermic introperitoneal chemotherapy (HIPEC) may improve the prognosis of PM in GC to some extent, but more clinical studies are warranted (1). These findings emphasize the importance of the quest for novel biomarkers for early diagnosis and provide new therapeutic strategies.

The mechanism of PM in GC remains unclear. According to the hypothesis of “Seed and Soil,” the pathogenesis of PM in GC involves shedding cancer cells from the primary site. Cancer cells survive in the abdominal cavity and adhere to the peritoneum. Cancer cells subsequently invade the subperitoneal tissue, proliferation, and angiogenesis (24). The TME plays an important role in PM. The TME has been recognized to regulate the proliferation, invasion, metastasis, and therapy in cancers (31). Hypoxia, low glucose levels, and ascites are often present in the abdominal cavity. The premise for the PM in GC is that the shed cancer cells can survive in hypoxia and the low-glucose microenvironment. The precise role of hypoxia in PM of GC is unclear and needs further investigation. FMOs act as phase I metabolic enzymes in the liver and play an important role in oxygen-related metabolism. Notwithstanding that the relationship between the FMO family and cancer has drawn much attention, the correlation between the FMO family and PM in GC has not been investigated. To our knowledge, this is the first study to report the role of the FMO family in GC-related PM.

FMO families are closely related to the occurrence, development, therapy, and prognosis of various cancers. Moreover, FMOs have been associated with the metabolism of multiple chemotherapy drugs (32–34). A study revealed that the overexpression of FMO1 independently predicted favorable recurrence-free survival (RFS) in papillary thyroid (18). Moreover, FMO2 was upregulated in the epithelial ovarian cancer tumor stroma and correlated with fibroblast activation. The results showed that FMO2 was associated with immune components, suggesting that it may be a prospective target for immunotherapy (35). It has been confirmed that FMO4 expression was decreased in hepatocellular cancer (HCC) tissue, and the low expression of FMO4 was a negative biomarker for HCC. FMO4-related bile acid metabolism is essential for immunotherapy in HCC. Accordingly, FMO4 may be a prognostic marker and potential therapeutic target in HCC (21). The overexpression of FMO5 was found in colorectal cancer (CRC) tissue, and FMO5 served as an independent prognostic factor for the survival of CRC (22).

In this study, we found that the expression of FMO1 was upregulated in GC tissues, while FMO2 and FMO4 were lowly expressed. Accordingly, the expression of the FMO family may correlate with tumorigenesis in GC. It is widely thought that FMO1 may promote tumorigenesis in GC. We further analyzed the correlation between the FMO family and the clinicopathological characteristics of GC. The expression of FMO1 and FMO2 was increased in patients with advanced-stage disease. FMO1 and FMO2 were positively correlated with the T stage. In addition, the expression of FMO2 was significantly increased in the N3 stage compared with N0. However, there was no significant difference in the expression of the FMO family between primary cancer and distant metastasis. In contrast, high FMO1 expression was associated with unfavorable RFS (HR: 1.112, CI:1.002–1.223, *p =* 0.045). Therefore, it can be inferred that the FMO family is associated with the development of GC, and FMO1 and FMO2 play a significant role in advanced-stage GC.

As shown above, the expression of FMO1 and FMO2 increased significantly in the advanced GC, but there was no significant correlation between distant metastasis and expression, which may be attributed to the low proportion of STAD patients with distant metastasis. Furthermore, FMO1 and FMO2 expression correlated significantly with the T stage. According to TNM staging, the T stage was mainly related to the depth of GC invasion. Indeed, T4 stage disease indicates that GC cancer cells have infiltrated the serosa of the stomach. It has been confirmed that serosa invasion is an independent risk factor of PM in GC. Therefore, we speculated that FMO1 and FMO2 are related to PM of GC. GSE15081 dataset analysis revealed that FMO1 was significantly upregulated in GC patients with PM. Both FMO1 and FMO2 correlated with PM in GC, although a more robust correlation was found with FMO2. Hence, the FMO family is important in regulating PM in GC.

PM is the most frequent mode of metastasis in GC, which differs from hematologic and lymphatic metastasis. The treatment of PM is different from distant metastasis in GC. Accurate identification of high-risk GC patients with PM can improve the patient’s prognosis and quality of life. The mechanism of PM in GC is unknown, with no clinical prediction model currently available for clinical practice. Many prediction models have been established to predict PM in GC from multiple perspectives, but these models have yielded insufficient prediction or poor practicability. We constructed a 10-gene panel model with satisfactory predictive performance (AUC = 0.932), including FMO1 and FMO2. To facilitate the application of the model, we established a 7-gene panel model (AUC = 0.927), which contains FMO2. The model yielded a good prediction performance and practicability. To facilitate the model’s application, we constructed a nomogram with FMO2 expression. Given that the FMO family is closely related to PM in GC, the model based on the FMO family members can effectively predict the PM risk.

The “Seed and Soil” theory can describe PM in GC to some extent, but further research is warranted to understand the exact mechanism. The FMO family is mainly known as a drug metabolism enzyme in the liver. An increasing body of evidence suggests that the FMO family is associated with tumorigenesis, metastasis, and treatment in many cancers. As mentioned above, the FMOs family plays a key role in PM development in GC. To further explore the potential mechanism of the FMO family in regulating PM in GC, we conducted GO and KEGG analyses. Overwhelming evidence substantiates that the FMO family promotes PM of GC by regulating the TME. Both FMO1 and FMO2 regulate the expression of collagen. As previously reported, the nomogram based on collagen can predict postoperative PM in GC with serosal invasion (25). Accordingly, the mechanism of the FMO family in promoting PM in GC is mainly by regulating the TME.

Immunotherapy is an important therapeutic strategy based on surgery, systematic chemotherapy and targeted therapy in GC (8). In 2021, nivolumab combined with systemic chemotherapy has been recommended as a first-line therapy for unresectable or recurrent GC in Japan (36). Immunotherapy may be a new effective treatment for PM in GC. Following the NCCN guidelines, immunotherapy should be recommended for advanced gastric cancer patients with MSI-H, dMMR, EB (+) or CPS>1 (37). However, current molecular markers exhibit limited performance in accurately predicting the prognosis of immunotherapy in GC. In the present study, we investigated the relationship between the expression of FMO1, FMO2 and immune infiltration. The results showed an intricate relationship between FMO1 and FMO2 expression and infiltration of multiple immune cells. Accordingly, FMOs may serve as potential markers for immunotherapy in GC.

However, the limitations found in this study should be acknowledged. Given that this study is a retrospective study based on public databases (TCGA and GSE1805), the analyzed clinical information was not complete. Moreover, our results have not been verified *in vivo* and *in vitro*. Finally, the mechanism of the FMO family members in regulating PM in GC needs to be further investigated.

## Conclusion

FMOs serve as novel markers and potential therapeutic targets for PM in GC. The clinical prediction model based on the FMO family exhibited a satisfactory performance in predicting PM in GC patients. Given that the FMO family members regulate the TME, they represent potential therapeutic targets in this patient population.

## Data availability statement

The datasets presented in this study can be found in online repositories. The names of the repository/repositories and accession number(s) can be found in the article/**Supplementary Material**.

## Author contributions

The corresponding authors, LZ and FH, designed the study; DH, SZ, and JT acquired and analyzed the data; XG, GZ, BY, and LX drafted the manuscript. All authors contributed to the article and approved the submitted version.
