# Supplementary material for: FMO family may serve as novel marker and potential therapeutic target for the peritoneal metastasis in gastric cancer
Source: Front Oncol. 2023 May 18;13:1144775. doi: 10.3389/fonc.2023.1144775 (PMC10234505; doi:10.3389/fonc.2023.1144775)
Supplement: Supplementary file 1 [file DataSheet_1.docx]

**FMO family serve as novel marker and potential therapeutic target for the Peritoneal metastasis in gastric cancer**

Xumeng Gong^a b†^, Dong Hou^d†^, Shengning Zhou^d^, Jianan Tan^d^, Guangyu Zhong^d^, Bing Yang^d^, Lang Xie ^c^, Fanghai Han ^d^*****, Lin Zhong^d^*****

^a^ Department of Surgical Oncology, Yuebei people`s hospital, Shaoguan, Guangdong 512205, China;

^b^ Department of Head-Neck and Breast Surgery, Yuebei People’s Hospital of Shantou University, Shaoguan, Guangdong, 512205, China;

^c^ Department of General surgery, Zhujiang Hospital, Southern Medical University, Guangzhou, Guangdong 510280, China;

^d^ Department of Gastrointestinal Surgery, Sun Yat-sen Memorial Hospital, Sun Yat-sen University, Guangzhou, Guangdong 510120, China.

***Corresponding author**

Zhonglin7@mail.sysu.edu.cn (Lin Zhong)，[hanfh@mail.sysu.edu.cn](mailto:hanfh@mail.sysu.edu.cn) (Fanghai Han)

^†^ These authors made equal contributions to this work.


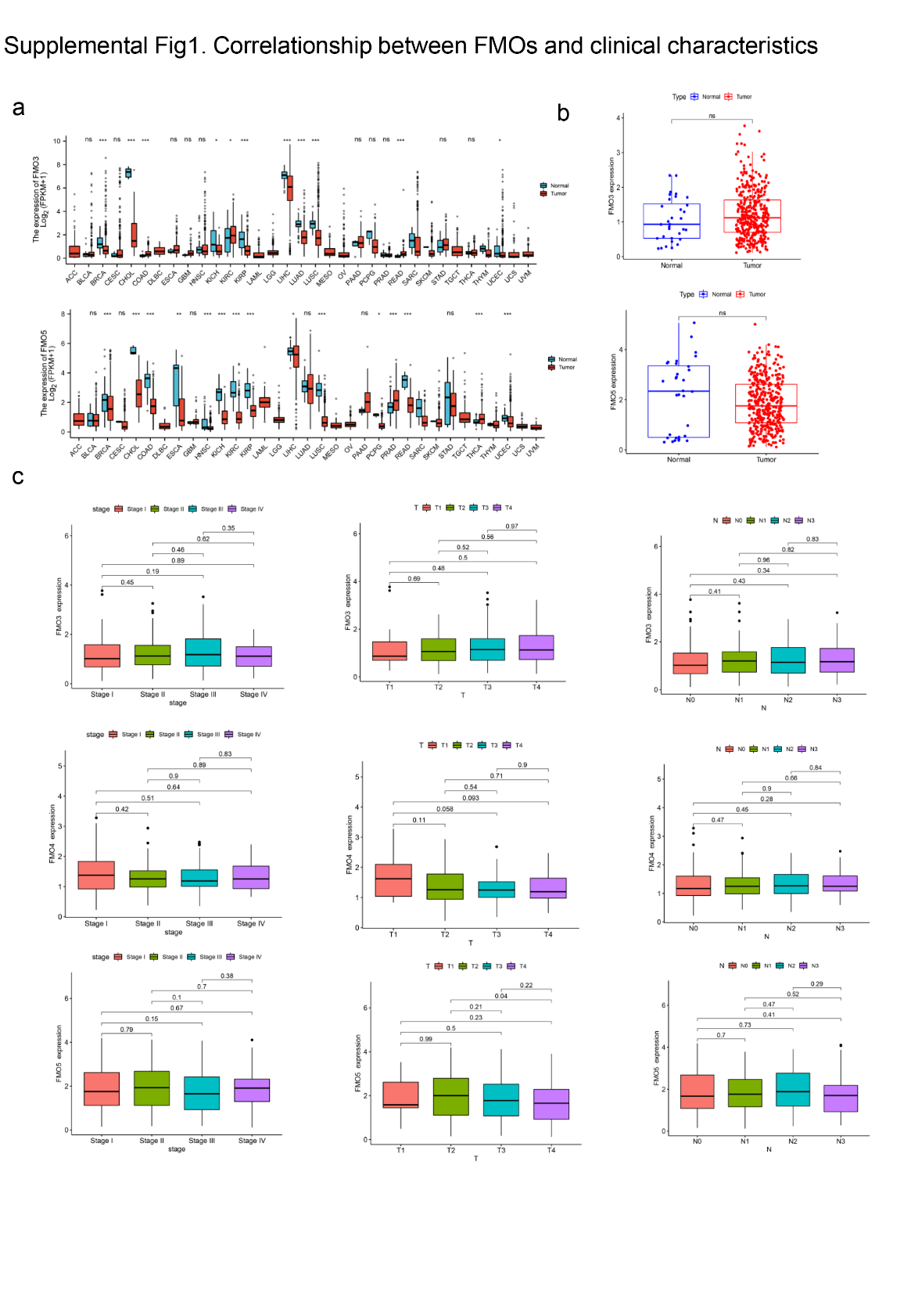


**Supplemental Fig1. Correlation between FMOs and clinical characteristics.** a. The expression of FMO3 and FMO5 in different cancers from TCGA data analyzed. b. FMO3 and FMO5 expression in normal and tumor tissues in GC from TCGA data. c. Association between FMO3, FMO4, FMO5 expression and the pathological, T, and N stage of GC.


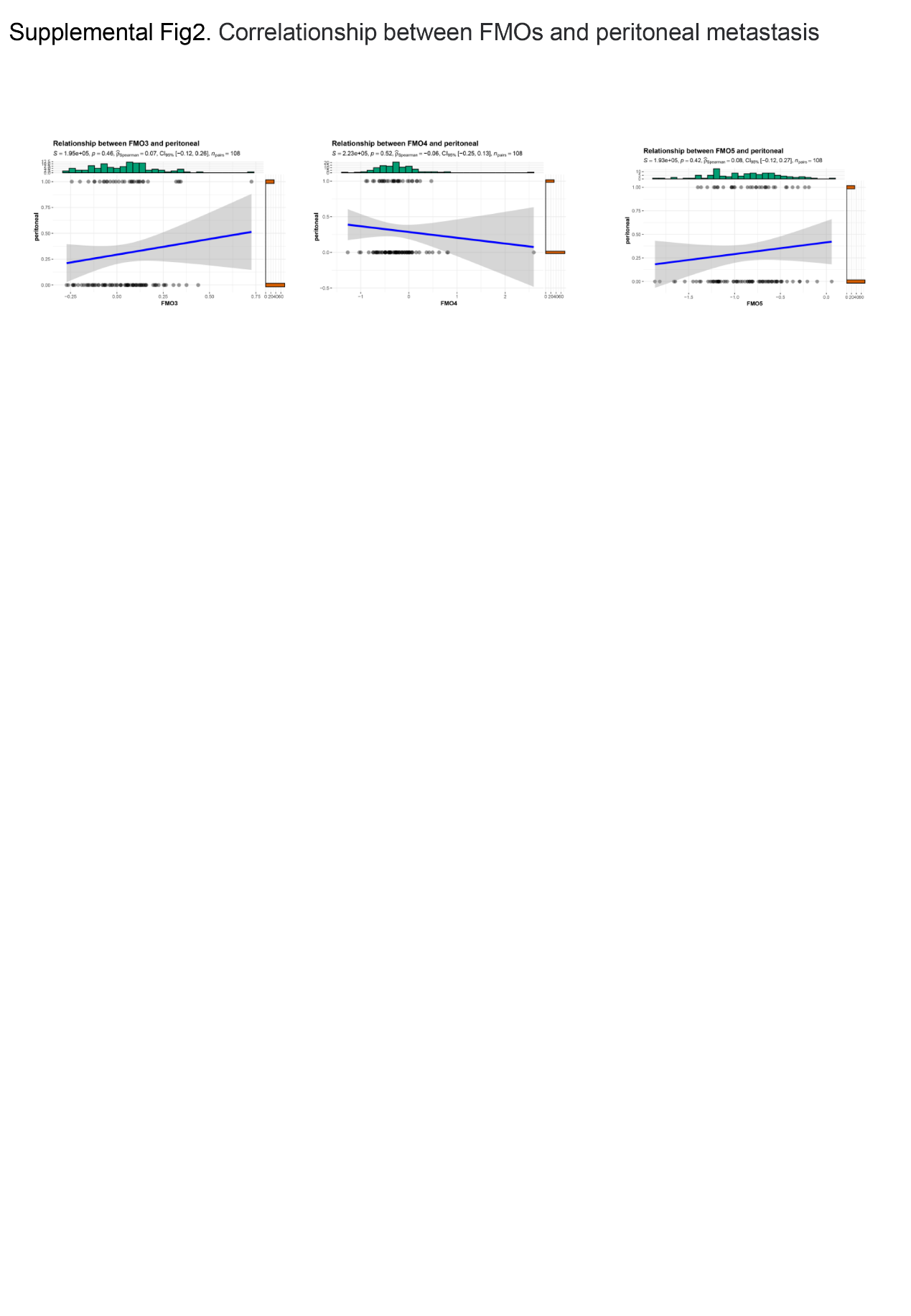


**Supplemental Fig2. Correlation between FMOs and clinical PM.** The association between FMO3, FMO4, FMO5 and PR.


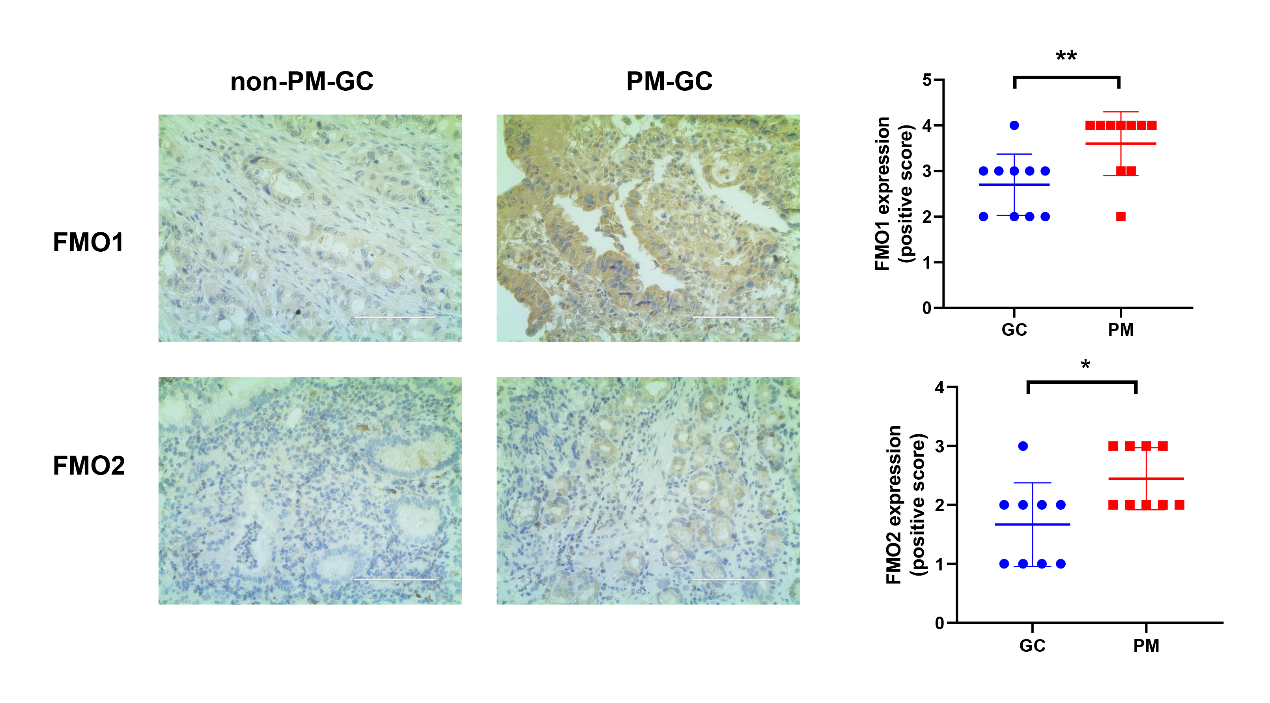


**Supplemental Fig3. The expression of FMO1 and FMO2 in non-PM-GC and PM-GC patients.** IHC staining for FMO1 and FMO2 expression in non-PM-GC and PM-GC tumor tissues. Representative images were shown (scale bar, 100μm). (Student’s 2-tailed t test, *, *p* <0.05; * *, *p<* 0.01).


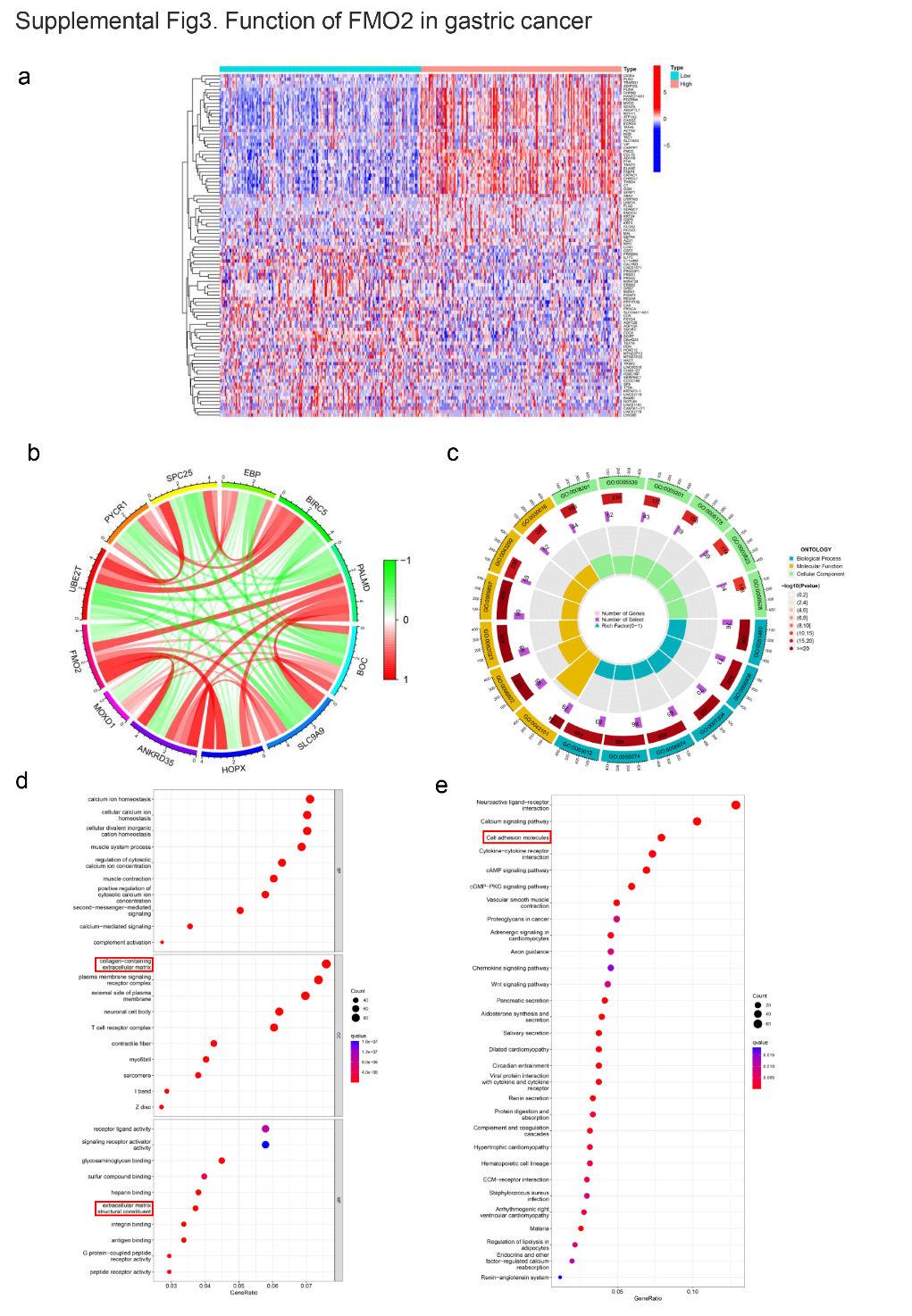


**Supplemental Fig4. The potential mechanism of FMO2 regulate PM in GC.** a. The differentially expressed genes between the high FMO2 group and the low FMO1 group, the results were represented by heatmap. b. Chord diagram was constructed, based on FMO2and co-expression genes. The red line indicated positive regulation, while the blue indicated negative regulation. c. GO enrichment analysis was shown by circle diagram. The outermost circle indicates the GO ID, the middle circle indicates the number of genes in the GO term, the innermost circle represents the DEGs in the GO term, and the bar graph of the circle diagram represents the ratio of genes in the GO term. d. GO analysis of the DEGs related to FMO2. The results are shown in a bubble plot. The size of the bubble represents the number of genes, and the color of the bubble represents the adjusted *p* -value. e. KEGG analysis of the DEGs related to FMO1. The size of bubble represents the number of genes, and the color of the bubble represents the adjust *p* value.
